# Use of Thermoluminescence Dosimetry for QA in High-Dose-Rate Skin Surface Brachytherapy with Custom-Flap Applicator

**DOI:** 10.3390/s23073592

**Published:** 2023-03-30

**Authors:** Francesco Manna, Mariagabriella Pugliese, Francesca Buonanno, Federica Gherardi, Eva Iannacone, Giuseppe La Verde, Paolo Muto, Cecilia Arrichiello

**Affiliations:** 1 Department of Physics “E. Pancini”, Federico II University, 80126 Naples, Italy; 2 Centro Servizi Metrologici e Tecnologici Avanzati, Federico II University, 80146 Naples, Italy; 3 National Institute of Nuclear Physics, Section of Naples, 80126 Naples, Italy; 4 Radiotherapy Unit, Istituto Nazionale Tumori, Istituto di Ricovero e Cura a Carattere Scientifico (IRCCS), Fondazione G. Pascale, 80131 Naples, Italy

**Keywords:** surface brachytherapy, thermoluminescent dosimeter, custom applicator, quality assurance, radiation dosimetry, in vivo dosimetry

## Abstract

Surface brachytherapy (BT) lacks standard quality assurance (QA) protocols. Commercially available treatment planning systems (TPSs) are based on a dose calculation formalism that assumes the patient is made of water, resulting in potential deviations between planned and delivered doses. Here, a method for treatment plan verification for skin surface BT is reported. Chips of thermoluminescent dosimeters (TLDs) were used for dose point measurements. High-dose-rate treatments were simulated and delivered through a custom-flap applicator provided with four fixed catheters to guide the Iridium-192 (Ir-192) source by way of a remote afterloading system. A flat water-equivalent phantom was used to simulate patient skin. Elekta TPS Oncentra Brachy was used for planning. TLDs were calibrated to Ir-192 through an indirect method of linear interpolation between calibration factors (CFs) measured for 250 kV X-rays, Cesium-137, and Cobalt-60. Subsequently, plans were designed and delivered to test the reproducibility of the irradiation set-up and to make comparisons between planned and delivered dose. The obtained CF for Ir-192 was (4.96 ± 0.25) μC/Gy. Deviations between measured and TPS calculated doses for multi-catheter treatment configuration ranged from −8.4% to 13.3% with an average of 0.6%. TLDs could be included in clinical practice for QA in skin BT with a customized flap applicator.

## 1. Introduction

High dose rate (HDR) surface brachytherapy (BT) is nowadays the treatment of choice for cutaneous lesions when the risk of cosmetic or functional side-effects of surgery or external beam radiotherapy is high [1,2].

Technological evolution has made BT a faster and more accurate therapy over the years, especially with the implementation of modern 3D imaging modalities such as computed tomography (CT) and magnetic resonance (MR) [3,4,5,6,7,8,9].

However, despite these major advances, the risk of errors in HDR BT planning, as well as in delivery (mainly due to manual procedures), is high compared to other forms of radiation therapy [10]. Some of reported errors are: (i) erroneously connected transfer guide tubes; (ii) inaccurate applicator/catheter positioning; (iii) wrong measurements of catheter lengths, and (iv) treatment planning system (TPS) data entry errors such as incorrect specified source strength or wrong treatment plan approved or imported [11,12].

To prevent such errors and assure patients safety, codes of practice and recommendations for quality assurance (QA) of HDR BT have been published [13,14,15,16,17]. However, the existing protocols do not completely cover HDR surface BT because of its unique geometric, scatter, and energy features related to the use of surface applicators. As a consequence, treatments are usually delivered without independent verification [18].

Although dosimetric studies are available for some type of applicators, such as the H-type Leipzig and Valencia from Nucletron [19,20,21], the Varian surface applicator set [22] and electronic BT applicators [23,24,25], systematic reviews and meta-analysis regarding these kinds of treatments are lacking in the literature [26], especially concerning custom molds and flaps applicators that are widely used for skin treatments [27,28,29].

At present, the formalism introduced by the American Association of Physicists in Medicine (AAPM) Task Group No. 43 Report (TG-43) [30] and the updated report [31] is the standard for dose calculation in clinical TPSs [32]. It assumes that the patient is totally made of water, therefore neglecting tissue inhomogeneities and the presence of non-water equivalent mediums as air cavities and applicator materials.

Skin treatments are characterized by the absence of radiation intersource attenuation and of full scatter conditions, but even if there are studies supporting the use of TG-43 in medical practice [33,34,35], deviations between prescribed and actually delivered doses may be clinically relevant.

This encourages, where available, the use of model-based dose calculation algorithms (MBDCAs) with the recommendations given by Report of AAPM TG-186 [36]. MBDCAs offer the possibility of departing from full-water geometries by modeling radiation transport in non-water media (tissues, applicators, air-tissue interfaces), resulting in a much more physically accurate reconstruction of the dose distribution delivered to the patient.

The aim of this work was to develop a reproducible and fast method that could be applied to clinical practice for dosimetric QA in a skin surface BT plan.

Dosimetry was performed by using thermoluminescence dosimeters (TLDs), which are proven to be high-performance tools for in vivo dosimetry (IVD) and QA in radiation therapy [37,38,39,40,41,42,43,44,45,46]. Comparisons with results obtained by Monte Carlo (MC) simulations and other dosimetry methods (e.g., ionizing chambers or gafchromic films) showed good agreement in HDR BT treatments [13]. However, TLD chips are the only available dosimeters allowing to perform point dose measurements, and only a few studies are published regarding their specific use in skin surface BT.

Skin BT treatments were simulated and delivered on a phantom with a catheter-based custom flap applicator, and comparisons between measured and calculated dose distributions were made.

## 2. Materials and Methods

TLDs-100 were calibrated to Ir-192 to use them as dosimeters for skin surface BT treatments. The TLD analyzing system is available at the Physics Department, University of Naples Federico II.

At the Radiotherapy Department of Istituto Nazionale Tumori G. Pascale, skin treatments were performed with customized flaps holding up catheters. This applicator was then fixed on a patient-specific thermoplastic mask to guarantee reproducibility, and the treatment was planned with a multi-catheter technique. Following this procedure, treatments with active dwell positions at the tip of catheters were planned and delivered on a phantom simulating the patient. TLDs were used to test the accuracy of the reconstruction method, and the rightness of channel-mapping and connections, by comparing measured and TPS calculated doses.

### 2.1. TLD-100 Dosimetry System

TLDs used in the study were Thermo Scientific™ LiF:Mg,Ti provided by Harshaw Chemical Company. A batch of 20 chips of 3.2 × 3.2 × 0.89 mm^3^ size, 2 mm spatial resolution and 2.64 g/cm^3^ density were used. They have an effective atomic number Z_eff_ = 8.2, which is close to the one of soft tissues, which is 7.64 [47], very low fading (5% per year), and a wide measurement dose range (10 pGy-10 Gy). Each dosimeter has its own identification number (ID).

TLD reading was performed with a Thermo Scientific™ Harshaw TLD™ Model 3500 Manual Reader provided by Harshaw Chemical Company. The reader is equipped with pure (99.995%) nitrogen supply needed to suppress chemiluminescence and spurious signals not related to irradiations. Readout was performed after a 10 s pre-heating to 100 °C by integrating the thermoluminescent signal up to 300 °C with a linear heating rate of 5 °C/s (40 s total integration time) chosen to optimize the signal-to-background ratio [48]. Measurements were obtained by integrating the signal in the region 150–250 °C, where the main peak is (at 195 °C), obtaining the reading R.

Annealing was performed in air with the TLD Annealing Oven “TLD Heat” provided by RadPro.

The pre-irradiation annealing procedure consisted of: 1 h heating at 400 °C, room temperature cooling, 2 h heating at 100 °C, and room temperature cooling [49] with programmed ramps of 10 min.

Characterization of dosimeters was made by evaluating each individual sensitivity factor and then calibrating to Ir-192′s average photons energy (E_average_=380 keV) [50,51].

#### 2.1.1. TLD-100 Dosimetry System

**Figure 1:**
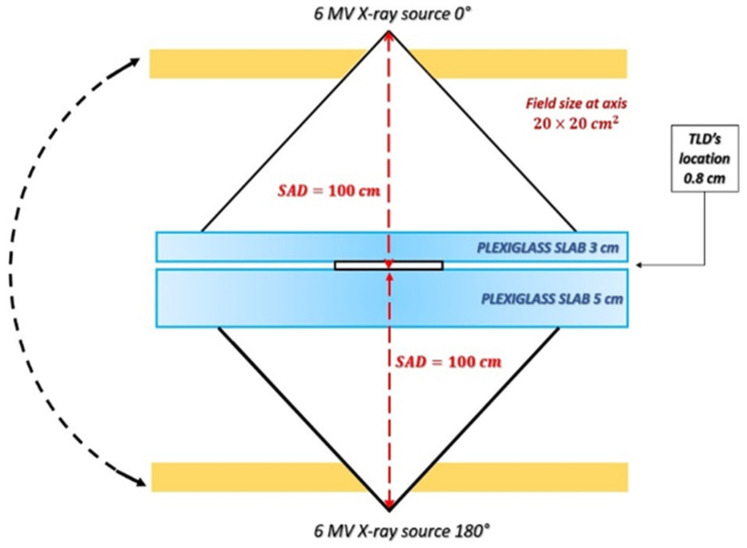
Irradiation set-up to 6 MV photons. Plexiglass slabs of 3 and 5 cm thickness were added to take into account the build-up phenomenon and backscatter radiation to deliver a uniform dose to targets. TLDs were put into a water-equivalent dish of 0.8 cm thickness. The radiation field was a photon beam with the size of 20 × 20 cm^2^ at a Source-to-Axis Distance SAD = 100 cm.

All TLDs were exposed to a uniform dose distribution of 2 Gy computed on Monaco TPS by Elekta with a collapsed cone convolution (CCC) algorithm and produced by a pair of opposing 6 MV photon beams. The reference set-up is shown in Figure 1. Irradiations were performed with Elekta Synergy Linear Accelerator (Elekta Instrument AB Stockholm).

#### 2.1.2. Calibration to Ir-192′s Energy

Since TLD-100 response varies significantly with radiation energy, to perform accurate dosimetry for BT treatments with Ir-192, calibration was performed indirectly by exposing the dosimeters to three different radiation fields:

- 250 kV X-ray spectrum with 1 mm Cu added filtration (HVL = 2.1 mm Cu) with a Siemens Stabilipan 2 X-ray tube (E_average_ = 114 keV obtained with software TASMICS [52]).
- Cesium-137 (Cs-137) with Gammacell^®^ 40 Exactor by Best Theratronics (E_average_ = 662 keV).
- Cobalt-60 (Co-60) with the Gammacell^®^ 220 provided by Atomic Energy of Canada Limited (E_average_ = 1250 keV).

Ir-192 was not used for calibration because accurate dose delivery in positions close to the source is made challenging by steep dose gradients [53,54].

The batch of 20 TLDs was randomly divided into four groups of 5 dosimeters. One group was used for radiation background, while the remaining groups were irradiated with known radiation doses of 0.5, 1.5, and 5 Gy, with each one being exposed to the three radiation fields. For each exposure, a graph of reading vs dose was plotted and, through a linear fit the calibration factor (CF), was evaluated as the slope of the line.

A graph of CF vs energy was then plotted and the factor for Ir-192 was obtained as linear interpolation [55,56,57].

### 2.2. BT Treatment Planning and Irradiations

**Figure 2:**
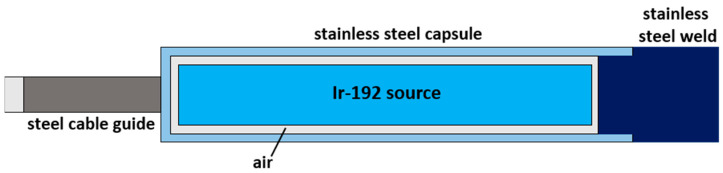
Schematic representation of the Iridium-192 Flexisource (adapted from Granero et al. [58]). The Ir-192 core is 3.50 mm long and has a diameter of 0.60 mm.

Skin BT treatments were driven by the remote afterloader Flexitron (Elekta Instrument AB Stockholm), which guides the Isodose Control HDR Ir-192 (model “Flexisource”, Veenendaal, The Netherlands) source in the catheters. A schematic representation is shown in Figure 2.

**Figure 3:**
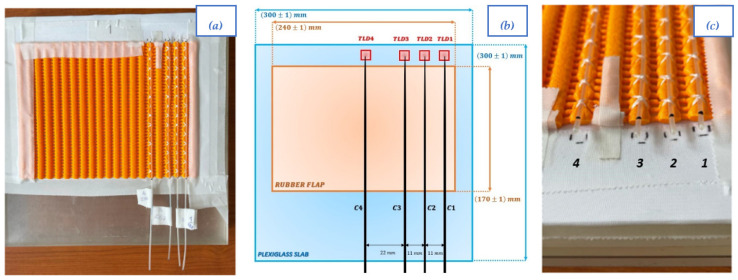
View from above (**a**), schematic representation of the same view with dimensions, where red squares indicate the position of the TDLs, (**b**) and front view (**c**) of the surface applicator and the phantom. Catheters were labelled as C1, C2, C3 and C4 as well as TLDs as shown.

Treatments were simulated on a phantom with a custom applicator. A rubber flap was fixed above plexiglass slabs with a total thickness of 4 cm. As shown in Figure 3, four catheters were attached on the flap, and the catheter’s tips were properly marked on phantom surface to maintain the correct placement of TLD chips beneath catheter’s tips.

Catheters were labelled as C1, C2, C3, and C4, in addition to the TLD positions, as shown in Figure 3.

Given the dimensions and geometry of the applicator, activating one single dwell position at the tip of C1 or C4 allows to perform dose measurements in positions that are 4.5 to 44.0 mm distant from the source.

The CT-based planning was elaborated on the TPS Oncentra Brachy by Elekta, which integrates TG-43 formalism for dose calculation, on images acquired with Toshiba’s Aquilion Large Bore (LB) CT system.

Two plans, each with a single dwell position at the tip of the catheters, were elaborated with the purpose of first verifying the reproducibility of the set-up and, instead, testing the response of dosimeters in the steep dose gradient region.

Furthermore, two additional plans, each in a multi-catheter configuration, were elaborated in order to achieve dose distributions that were as uniform as possible for TLD exposures below the tips of catheters.

In agreement with a typical fractional dose for a HDR skin BT treatment, the prescribed dose was chosen to be 4 Gy

Comparisons between measured dose values and TPS computed doses D_50_ were conducted to evaluate the actual dose distribution with respect to the expected one.

#### 2.2.1. Plans with a Single Dwell Position

**Figure 4:**
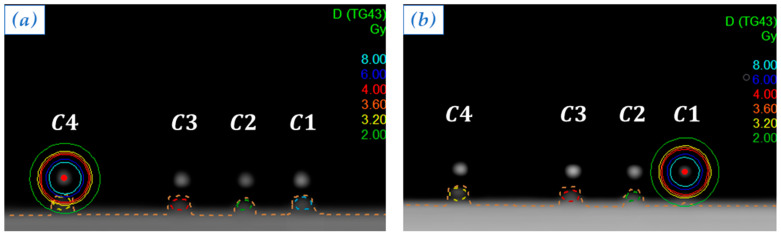
Isodose curves obtained by optimizing the plan with a single active dwell position in C4 (**a**) and the plan with a single active dwell position in C1 (**b**). Each color corresponds to a dose value, as reported in the legend.

In order to study TLD’s response at different distances from the radioactive source actual position, in the first two plans (Figure 4), dwell positions were chosen to be C4 and C1.

Both plans were delivered five times to test the set-up reproducibility.

Optimized isodose curves for the two plans are shown, respectively, in Figure 4a and in Figure 4b.

Both plans are computed on a 3 mm CT slice thickness in axial direction.

#### 2.2.2. Plans with Four Dwell Positions

**Figure 5:**
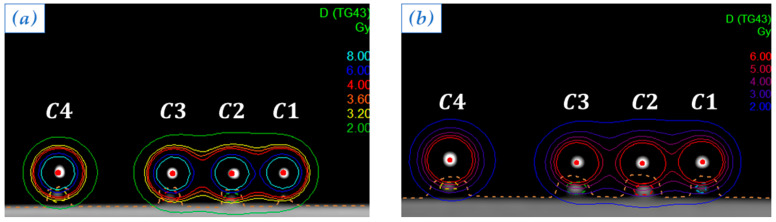
Isodose curves obtained by optimizing the 3 mm slice thickness plan with four active dwell positions (**a**) and the 1 mm slice thickness plan with four active dwell positions (**b**). Each color corresponds to a dose value, as reported in the legend.

The first multi-catheter plan was computed on a 3 mm CT slice thickness in the axial direction and was delivered five times to perform dose measurements. Optimized isodose curves are shown in Figure 5a.

The second one is computed on a 1 mm CT slice thickness in the axial direction and was delivered twice. Images of minor slice thickness allows a better location of TLD, improving the accuracy in dose delivery. Optimized isodose curves are shown in Figure 5b.

## 3. Results

### 3.1. TLD-100 Characterization

Results from previously discussed irradiations for TLD characterization are presented.

#### 3.1.1. Sensitivity Factors

After being exposed to a uniform dose of 2 Gy, each dosimeter of the batch was read (*R_i_*). The arithmetic mean $\bar{R}$ was calculated, and each sensitivity factor was evaluated as:

(1) $$\;S_{i}=\frac{R_{i}}{\bar{R}}\;\forall \;i\in 1,\ldots ,\;20$$

Measured sensitivity factors ranged between 0.85 and 1.12. No dosimeter was rejected; given the reduced number of TLDs, we accepted a range of sensitivity factors from 0.80 to 1.20, as advised by Plato and Miklos [59].

#### 3.1.2. Calibration Factor for Ir-192′s Energy

For each exposure, every reading value *R_i_* was corrected using the corresponding sensitivity factor *S_i_*. Then the arithmetic mean of the five readings of background radiation ${\bar{R}}_{BG}$ was evaluated and subtracted from each measure to obtain the net value for each TLD $R_{i}^{net}$:

(2) $$\;\;R_{i}^{net}=\frac{R_{i}}{S_{i}}-{\bar{R}}_{BG}$$

where

(3) $${\bar{R}}_{BG}=\frac{1}{5}\cdot \overset{5}{\underset{j=1}{\sum }}\frac{R_{j}^{\left(BG\right)}}{S_{j}}$$

For each of the three subgroups of dosimeters exposed to the dose D, the arithmetic mean *R^net^* was calculated and plotted as a function of dose to obtain the calibration curve.

**Figure 6:**
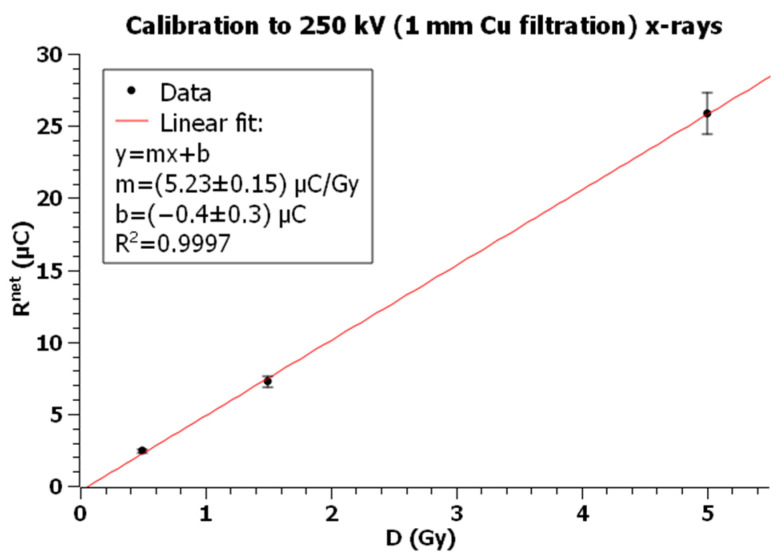
Plot of TLD net readings vs dose for exposures with 250 kV and 1 mm Cu filtration X-rays. Error bars represent the expanded uncertainty (*k* = 2) for X-rays calibration from Table 4.

**Figure 7:**
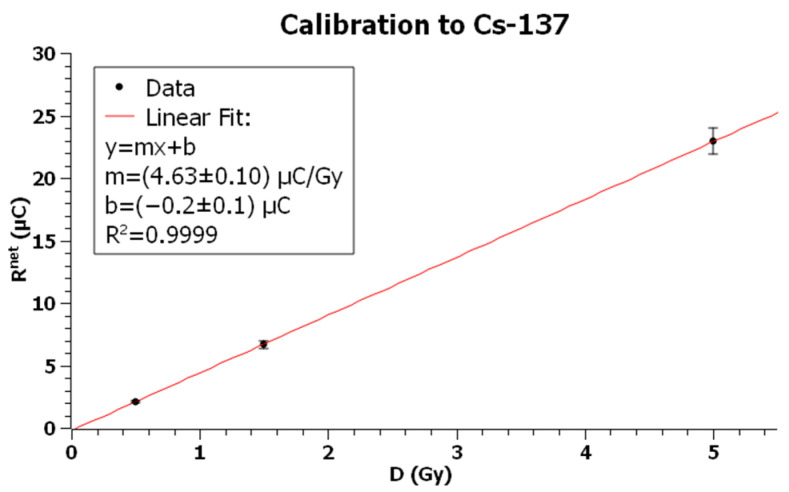
Plot of TLD net readings vs dose for exposures with Cs-137. Error bars represent the expanded uncertainty (*k* = 2) for Cs-137 calibration from Table 4.

**Figure 8:**
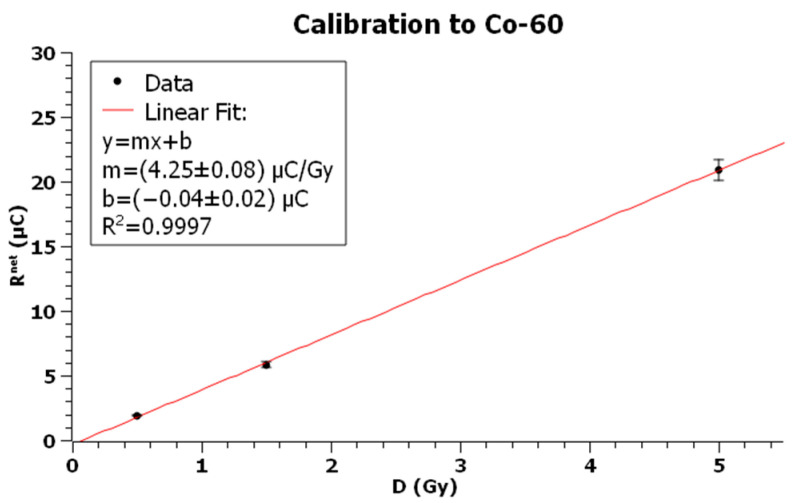
Plot of TLD net readings vs dose for exposures with Co-60. Error bars represent the expanded uncertainty (*k* = 2) for Co-60 calibration from Table 4.

Table 1, Table 2 and Table 3 show the net measured reading for each dose point when TLDs were exposed, respectively, to X-rays, Cs-137, and Co-60, while Figure 6, Figure 7 and Figure 8 show the corresponding plots with linear fits. The square of the Pearson correlation coefficient, R^2^, was calculated to evaluate the goodness of the fits. Uncertainty analysis for calibration is shown in Table 4, taking into account TLD reproducibility, air-kerma rate determination, TLD positioning, photomultiplier tube (PMT) linearity correction, field uniformity, and reader stability. The TLD reproducibility type A statistical uncertainty was estimated as the percent standard deviation of the mean of all TLD readings used for calibration. The type B uncertainty has been obtained by non-statistical procedures; the air-kerma rate determination component of the uncertainty came from measurements performed at University of Naples Federico II and Istituto Superiore di Sanità [60]. The uncertainty for PMT linearity correction was determined by comparing measurements with and without the correction applied and evaluating the supralinearity index [61,62]. Reader stability uncertainty was considered a negligible source of uncertainty and assumed to be included in TLD reproducibility [63,64].

Combined type A uncertainty is the square-root of the linear sum of squared standard type A uncertainty component, and the same definition applies for combined type B uncertainty. Total standard uncertainty is obtained as the square-root of the linear sum of squared combined type A and combined type B uncertainties and corresponds to uncertainty with a 68.3% confidence level (that is, the probability of the true value falling within the uncertainty range is 68.3%). Expanded uncertainty, u, is obtained by multiplying total standard uncertainty with a coverage factor k. *k* = 2 was chosen for a confidence level of 95.5%.

The *CF* for X-rays resulted in:

$$CF_{250kV\_Xrays}=\left(5.23\pm 0.29\right)\;\frac{\mu C}{\mathrm{Gy}}$$

The *CF* for Cs-137 resulted in:

$$CF_{Cs-137}=\left(4.63\pm 0.21\right)\;\frac{\mu C}{\mathrm{Gy}}$$

The *CF* for Co-60 resulted in:

$$CF_{Co-60}=\left(4.25\pm 0.16\right)\;\frac{\mu C}{\mathrm{Gy}}$$

Uncertainties in measured calibration factors are expressed as expanded combined uncertainty with *k* = 2.

**Figure 9:**
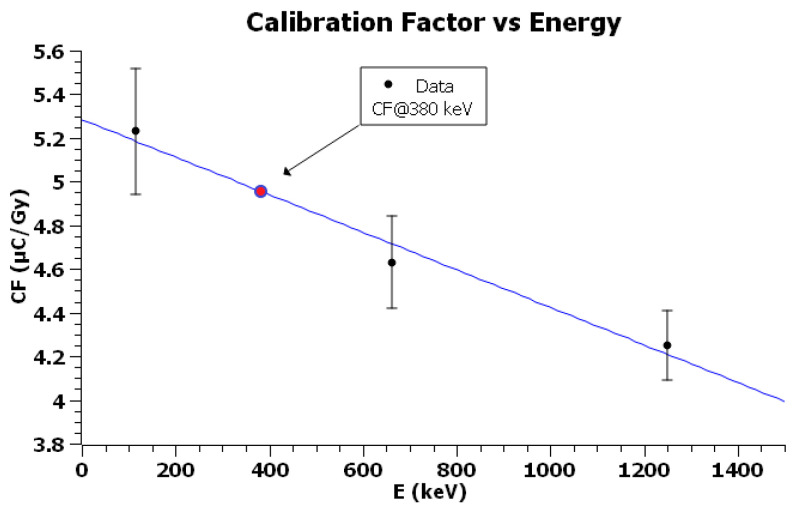
Plot of CF as function of average radiation energy. Error bars represent the total uncertainty (*k* = 2) for the TLD calibration measurements from Table 5.

The factor at 380 keV was obtained through linear interpolation on plot of *CF* as a function of average radiation energy E (see Figure 9). Data are shown in Table 5, and the interpolated factor with a relative standard uncertainty (*k* = 2) equal to 5.06% resulted in:

$$CF_{Ir-192}=\left(4.96\pm 0.25\right)\;\frac{\mu C}{\mathrm{Gy}}$$

### 3.2. Dose Measurements from BT Treatments

For each reading *R_i_*, the absorbed dose was calculated as:

(4) $$\;D_{i}=\frac{R_{i}}{S_{i}\cdot CF_{Ir-192}}$$

#### 3.2.1. Results from Exposures with a Single Active Dwell Position

Each plan was delivered four times, and the arithmetic mean of measured doses under the same catheter was calculated. The relative standard uncertainties, *u*(%), were evaluated by taking into account TLD reproducibility, PMT linearity correction, reader stability for the reading value *R_i_*/*S_i_*, and the uncertainties obtained from the interpolated CF to Iridium-192′s energy, as previously described. More specifically, since the uncertainty for the PMT linearity correction is greater for higher doses where larger corrections are applied, it was determined as 1.06% for TLDs receiving a prescription dose of 4 Gy and was chosen to be 0.10% for all other dosimeters receiving doses smaller than 1 Gy.

Comparisons were made between measured values by TLD and TPS calculated doses *D*_50_ by evaluating the difference:

(5) $$\;\Delta D\left(\%\right)=\frac{D_{m}-D_{50}}{D_{m}}\cdot 100$$

Since small geometric uncertainties can lead to very high dose discrepancies because of steep dose gradients, the reproducibility in TLD positioning was assessed by verifying that dosimeters placed in the same positions always measure the same dose within estimated uncertainties when the treatment is repeated in the same session. The average of the dose percentage differences between four subsequent exposures was calculated as:

(6) $$\;\bar{\Delta D}\left(\%\right)=\frac{1}{3}\cdot \overset{3}{\underset{i=1}{\sum }}\frac{\left|D_{i}-D_{i+1}\right|}{D_{i}}\cdot 100$$

Furthermore, to test the time reproducibility of the set-up, one further exposure was performed after t* = 1 week, and the percentage difference between *D_m_* and measured dose D_t*_ was evaluated as:

(7) $$\;\Delta D_{t^{*}}\left(\%\right)=\frac{\left|D_{m}-D_{t^{*}}\right|}{D_{m}}\cdot 100$$

Values are shown in Table 6 and Table 7 together with expanded combined uncertainty u on measured doses.

Estimated combined uncertainties ranged from 5% to 7% with an average of 6%.

The average percentage measured dose differences for subsequent exposures under the same catheter ranged from 0.4% to 2.8%.

The average percentage measured dose differences for exposures performed after 1 week ranged from 0.6% to 2.3%.

For all measurements, $\bar{\Delta D}<u$ and $\Delta D_{t^{*}}<u$.

Percentage difference between measured and calculated doses ranged from −40.5% to 17.5%. These minimum and maximum differences were observed in the proximity of Ir-192. Measurements performed at source-to-detector distances greater than 4.5 mm showed that the TPS overestimated the skin dose with discrepancies in the range of −12.4% to −2.6% with an average deviation of −7.2%.

#### 3.2.2. Results from Exposures with Four Active Dwell Positions

The 3 mm slice thickness plan was delivered five times, and the arithmetic mean of measured doses was calculated. The relative standard uncertainties, *u*(%), were evaluated, as previously described, by taking into account TLD reproducibility, PMT linearity correction, reader stability for the corrected reading value, and the uncertainties obtained from the interpolated CF. The comparisons between dose measurements and TPS calculated doses were evaluated by percentage difference (Equation 5).

**Figure 10:**
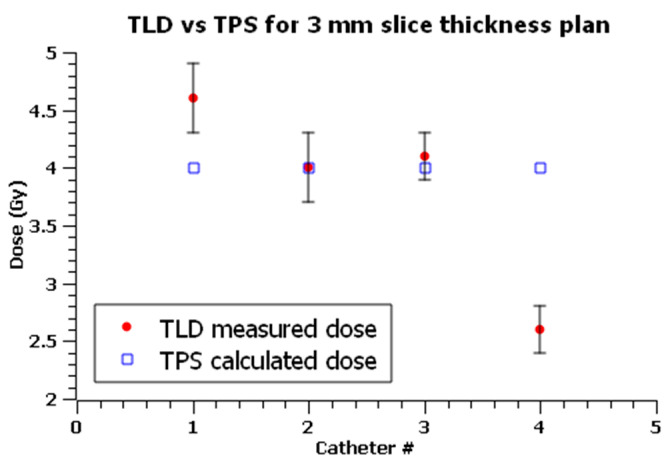
Comparisons between TLD measured doses (in red full dots) and TPS calculated doses (in blue empty squares) for the 3 mm slice thickness plan. x axis is the catheter number (#) corresponding to the point of measurement. Error bars show the estimated expanded (*k* = 2) uncertainty for TLD measurements.

Values are shown in Table 8, and Figure 10 shows a graphical comparison between the planned and measured doses in each position of the phantom, indicating agreement within the limits of uncertainties only for TLD2 and TLD3.

The same measurements and analyses were performed for the 1 mm slice thickness plan, which was delivered twice.

**Figure 11:**
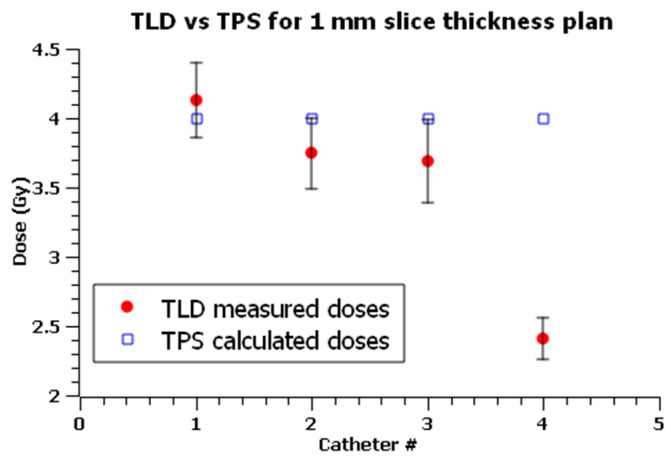
Comparisons between TLD measured doses (in red full dots) and TPS calculated doses (in blue empty squares) for the 1 mm slice thickness plan. x axis is the catheter number (#) corresponding to the point of measurement. Error bars show the estimated expanded (*k* = 2) uncertainty for TLD measurements.

Values are shown in Table 9, and Figure 11 shows a graphical comparison between the planned and measured doses in each position of the phantom, indicating agreement within the limits of uncertainties for all dosimeters but TLD4.

The 3 mm CT slices appear to be too large, considering TLDs dimensions, leading to less accurate locations of the dosimeters and, therefore, to possible systematic discrepancies between measured and TPS calculated doses. Nevertheless, results show agreement within estimated uncertainties for TLD2 and TLD3.

Instead, CT slices of 1 mm thickness allow a more accurate volume reconstruction of the dosimeters, leading to a more accurate dose delivery and a better agreement between the measured and calculated values, since TLD1′s measurement is also comparable to TPS calculated values.

However, measured dose values between the two plans for the corresponding TLDs are in accordance with the estimated uncertainties, highlighting the reliability of the results obtained from the 3 mm CT slice plan.

The estimated combined uncertainties, u, ranged from 6% to 8% with an average of 7%. Measurements performed under C4 showed a systemic overestimation of TPS up to −66.0%.

Excluding measurements under C4, discrepancies in measured and calculated doses ranged between −8.4% and 13.3% with an average of 0.6%.

## 4. Discussion

TLDs-100 were calibrated indirectly to Ir-192′s mean energy (380 keV) by linear interpolation of calibration factors measured for radiations of lower and higher energies. TLD response as function of energy in the range from about 10 keV to a few MeV has been investigated in the literature [65,66,67,68], and there are microdosimetric studies about the over-response of LiF:Mg,Ti relative to Co-60 [61,69].

Nunn et al. [65] and Tedgren et al. [66] measured the over-response of TLDs to Cs-137 relative to Co-60, obtaining 1.06±0.04 and 1.04±0.04, respectively (combined uncertainties with *k* = 2).

The value obtained in this work is $\frac{CF_{Cs-137}}{CF_{Co-60}}=1.09\pm 0.06$.

The agreement with prior literature estimates is fairly good within the limits of uncertainties.

Raffi et al. [70] previously determined the energy correction factor relating the TLD response to Ir-192 relative to Cs-137, following the methodology proposed by Nunn et al. [65]. They found an overresponse of 1.04±0.04 (combined uncertainty with *k* = 2).

The measured correction factor in this work is $\frac{CF_{Ir-192}}{CF_{Cs-137}}=1.07\pm 0.06$. The two values are consistent within the limits of uncertainties.

Calibration was performed experimentally, keeping into account the average energy of the photon spectrum emitted from the source, and was therefore not dependent on the irradiation geometry. The agreement with energy factors available in literature is good, despite the use of different LiF:Mg,Ti formulations or dimensions, different reader and/or annealing ovens, or different read-outs and/or annealing processes that affect the TLD response [67,71,72,73]. 

Improvements in calibration could be conducted by performing exposures to radiations of different average energies and looking for a comparison with the studies previously cited, or by reducing the estimated uncertainty; for example, more dosimeters could be used to reduce the type A uncertainty, and sources with a more accurate air-kerma rate determination could reduce the type B contribution.

In order to use TLD-100 for QA in skin BT, we performed exposures on a flat phantom and made comparisons between the measured and prescribed surface dose distributions. Treatment plans were realized by manual reconstruction and manual optimization, and delivered through a custom flap applicator with four catheters.

Two plans with one active dwell position at the tip of one catheter were delivered to test (i) the reproducibility of dose measurements at different distances from the source and (ii) the detector response in a steep dose gradients region.

Estimated expanded uncertainties u (with *k* = 2) ranged between 5% and 7%. For each source-to-detector distance, we evaluated the average discrepancy between 4 subsequent measurements, $\bar{\Delta D}$, finding that it is always smaller than the estimated uncertainties, meaning that TLDs placed in same positions always measure the same dose within estimated uncertainties. TLD measurements are reproducible in regions of steep dose gradients.

Reproducibility is also observed when delivery is repeated after t* = 1 week since the calculated discrepancy $\Delta D_{t^{*}}<u$ for each measurement, meaning that the TPS correctly takes into account the source decay and that the irradiation set-up is stable.

The percentage difference between measured and calculated doses ranged from −40.5% to 17.5%. The minimum and maximum differences were observed in the proximity of Ir-192 at a source-to-detector distance of 4.5 mm. This large discrepancy may be due to the dose gradient, which is very steep around the source and makes accurate measurements challenging. However, measurements performed at distances in the range of 12–44 mm showed that the TPS overestimated the skin dose, with discrepancies in the range of −12.4% to −2.6% with an average deviation of −7.2%.

We did not observe any correlation between the measured dose discrepancy and the source-to-detector distance, although it seems clear that, because of the steep dose gradient and the volume averaging effect in proximity of the source, TLDs are not an accurate tool to perform measurements when they are placed very close to an isolated dwell position.

Two plans with four active dwell positions, each at the tip of every catheter, were delivered to test the reproducibility of dose measurements in real skin treatment conditions.

The expanded uncertainty with *k* = 2 ranged from 6% to 8% with an average of 7%, which is consistent with prior literature estimates [74]. Even though the highest contribution to uncertainty was given by the CF, we expect to reduce the type A contribution by performing more repeated measures.

Measurements performed under catheter 4 showed maximum TPS overestimations of −53.3% and −66.0%.

Catheter 4 is relatively further away than the other three catheters, meaning that dose gradient issues are crucial; dose inhomogeneities inside TLD4 volume are more significative than at other positions, and a uniform dose distribution is more difficult to achieve.

However, real skin treatments are planned and delivered in a multi-catheter configuration that does not have isolated active dwell positions.

Excluding measurements under C4, dose deviations ranged between −8.4% and 13.3% with an average deviation of 0.6%.

The 1 mm CT slices allow a more accurate volume contouring compared to the 3 mm CT slices, leading to more reliable results, and, therefore, it is recommended to minimize slice thickness for accurate planning and delivery whenever using TLDs for dose measurements in HDR BT treatments.

The method is applicable for QA in multi-catheter skin surface BT since the measured dose distribution is, on average, in agreement with the prescribed one, even for measurements performed very close to the source.

We expect TLDs to be useful for different types of applicators as well as for more complicated dose distributions obtained with a higher number of active dwell positions, but more studies should be carried out. It would be beneficial to test the method in a not-flat geometry, for example, by using anthropomorphic phantoms of the head or other anatomical areas with curved surfaces.

Discrepancies between prescribed and measured doses are expected in surface treatments if using TG-43, since this formalism is appropriate only with full scatter conditions. Despite this, only few published studies are available about dosimetry in skin BT, especially regarding the use of custom flap applicators.

Fabiani et al. [75] and Raffi et al. [70] previously used TLDs to measure the exit skin dose in HDR partial breast BT, finding TPS overestimations of up to 47%, when measurements were performed on patients, and of up to 15% when performed on phantoms simulating treatment conditions.

While waiting for a standardized QA protocol for skin surface BT, and precise instructions for commissioning of custom flap applicators, the method proposed in this work may be used in a pre-treatment phase to significantly reduce errors due to manual procedures which are still required for this type of treatments. Verification of the treatment plan modality, dose, location, and number of active dwell positions, as well as verification of catheter length, labelling, and connections, are basic safety rules that should be implemented for skin BT in order to improve patient safety, and this can be achieved by the use of TLDs as described in the work. The only limitation in the use of TLDs is that the response can not be obtained immediately after irradiation, since the readout is always performed at least 24 h after exposure. However, if the treatment plans for patients are elaborated in advance, TLDs can still be implemented for routine QA procedures. In addition, despite the fact that the set-up time of placement may vary according to the complexity of the plan, it is on the order of 10 min, or even less if it involves a limited number of catheters and TLDs.

More specifically, we found that TLDs may not be used for dose measurements when placed very close to an isolated source because the steep dose gradients and volume averaging effects make accurate delivery challenging. However, if a more uniform dose distribution is achieved with close dwell positions, as it happens for ral skin treatment conditions, TLD-100 can reproduce the prescribed dose distribution even in the proximity of the source. Therefore, the use of TLDs can be a fast and valid method to test the accuracy of the reconstruction method and the rightness of channel-mapping, for example, by delivering plans with first dwell positions only.

## 5. Conclusions

This work showed the possibility of using TLD-100 for point dose measurements in surface BT treatments with Ir-192 delivered with a custom-flap applicator, determining their key role in integrating both QA and IVD in clinical routine.

Calibration to Ir-192′s energy was performed by linearly interpolating the CFs measured for greater and lower radiation energies. This indirect procedure allowed to avoid all the issues of a direct calibration to a HDR Ir-192 source, with the results being in agreement with the data available in the literature, making TLDs feasible for dosimetry in skin BT.

Exposures to plans with a single active dwell position proved the reproducibility of the method from set-up positioning to dose delivery, even when treatment delivery is repeated in time.

Results from real treatment condition exposures showed that the proposed method allows to evaluate the skin dose distribution in surface BT. Therefore, it can be used for independent verification of treatment planning.

Critical issues of dose measurements in BT are associated with the very steep dose gradients around the source because even small geometric uncertainties may lead to large dose discrepancies. Nevertheless, TLDs proved to be efficient dosimeters if a more uniform dose distribution inside the target is achieved by planning in a multi-catheter configuration; under these conditions, TG-43 formalism appears to be clinically suitable for target dose delivery in surface treatments, although more studies should be carried out, for example, with more complicate geometries and dose distributions and by using anthropomorphic phantoms or different applicators, or by making comparisons with MBDCAs.

In addition, TLDs can be useful to evaluate the dose delivered to OARs, where it is possible, to assess patient safety.

The need for a standard practice for QA in surface BT is still highly necessary to secure the most possibly accurate treatment outcome and reduce the risk of error.

## Figures and Tables

**Table 1:** Measured net values for each dose point when TLDs were exposed to 250 kV and 1 mm Cu added filtration X-rays spectrum. Relative uncertainties on $R^{net}$ are expressed in Table 4.

D (Gy)	$R^{net}\;(\mu C)\;$
** 0.5 **	2.416
** 1.5 **	7.243
** 5 **	25.84

**Table 2:** Measured net values for each dose point when TLDs were exposed to Cesium-137. Relative uncertainties on $R^{net}$ are expressed in Table 4.

D (Gy)	$R^{net}\;(\mu C)\;$
** 0.5 **	2.118
** 1.5 **	6.657
** 5 **	22.94

**Table 3:** Measured net values for each dose point when TLDs were exposed to Cobalt-60. Relative uncertainties on $R^{net}$ are expressed in Table 4.

D (Gy)	$R^{net}\;(\mu C)\;$
** 0.5 **	1.908
** 1.5 **	5.818
** 5 **	20.92

**Table 4:** Relative uncertainty analysis for TLD calibration to 250 kV X-rays, with 1 mm Cu added filtration, to Cs-137 and to Co-60.

		250 kV X-rays			^137^Cs			^60^Co	
**Uncertainty** **source**	Type A (%)		$\mathrm{Type}\;B\;\left(\%\right)$	Type A (%)		$\mathrm{Type}\;B\;\left(\%\right)$	Type A (%)		$\mathrm{Type}\;B\;\left(\%\right)$
**TLD reproducibility**	2.10			1.28			0.60		
**Air-kerma rate determination**			1.47			1.44			1.43
**TLD positioning**			0.10			0.10			0.10
**PMT linearity correction**			1.07			1.08			1.10
**Field uniformity**			0.10			0.10			0.10
**Reader stability**			0.01			0.01			0.01
**Combined**	2.10		1.85	1.28		1.81	0.60		1.81
**Total (*k* = 1)**		2.78			2.22			1.91	
**Expanded** **(*k* = 2)**		5.56			4.44			3.82	

**Table 5:** Measured CFs for different average radiation energies with estimated expanded uncertainties Δ*CF*.

$E\;\left(\mathrm{keV}\right)\;$	$CF\;\left(\mu C/\mathrm{Gy}\right)\;$	ΔCF (μC/Gy)
** 114 **	5.23	0.29
** 662 **	4.63	0.21
** 1250 **	4.25	0.16

**Table 6:** *D*_*m*_ over four subsequent exposures compared to TPS calculated dose *D*_50_ (left) and measured doses *D_m_* for a single exposure performed after 1 week (right) for the plan with a single active position in C4. The relative standard uncertainty *u*(%) with *k* = 2 is also shown along with percent differences.

Measures over Four Repeated Exposures						Measures after $t^{*}$	
Catheter	$D_{50}\;\left(\mathrm{Gy}\right)$	$D_{m}\;\left(\mathrm{Gy}\right)$	$u\left(\%\right)$	$\Delta D\left(\%\right)$	$\bar{\Delta D}\left(\%\right)$	$D_{t^{*}}\;\left(\mathrm{Gy}\right)$	$\Delta D_{t^{*}}\left(\%\right)$
** 1 **	0.050	0.048	6	−4.2	1.6	0.047	2.1
** 2 **	0.080	0.078	5	−2.6	0.4	0.077	1.3
** 3 **	0.1956	0.174	5	−12.4	2.3	0.170	2.3
** 4 **	4.0040	2.85	7	−40.5	2.7	2.79	2.1

t* = 1 week.

**Table 7:** *D*_*m*_ over four subsequent exposures compared to TPS calculated dose *D*_50_ (left) and measured doses *D*_*m*_ for a single exposure performed after 1 week (right) for the plan with a single active position in C1. The relative standard uncertainty *u*(%) with *k* = 2 is also shown along with percent differences.

Measures over Four Repeated Exposures						Measures after $t^{*}$	
Catheter	$D_{50}\;\left(\mathrm{Gy}\right)$	$D_{m}\;\left(\mathrm{Gy}\right)$	$u\left(\%\right)$	$\Delta D\left(\%\right)$	$\bar{\Delta D}\left(\%\right)$	$D_{t^{*}}\;\left(\mathrm{Gy}\right)$	$\Delta D_{t^{*}}\left(\%\right)$
** 1 **	4.0094	4.86	6	17.5	2.8	4.80	1.2
** 2 **	0.7609	0.701	7	−8.5	2.4	0.689	1.7
** 3 **	0.1956	0.177	6	−10.5	1.5	0.178	0.6
** 4 **	0.050	0.047	5	−6.4	0.8	0.048	2.1

t* = 1 week.

**Table 8:** $D_{m}$ compared to TPS calculated dose $D_{50}$ for the 3 mm slice thickness plan with four active dwell positions. The relative standard uncertainty $u\left(\%\right)$ with k=2 is also shown along with percent differences between planned and measured doses.

Catheter	$D_{50}\;\left(\mathrm{Gy}\right)$	$D_{m}\;\left(\mathrm{Gy}\right)$	$u\left(\%\right)$	$\Delta D\left(\%\right)$
1	4.0047	4.62	6	13.3
2	4.0026	4.00	6	−0.1
3	4.0017	4.11	6	2.6
4	4.0024	2.61	6	−53.3

**Table 9:** D_m_ compared to TPS calculated dose D_50_ for the 1 mm slice thickness plan with four active dwell positions. The relative standard uncertainty *u*(%) with k = 2 is also shown along with percent differences between planned and measured doses.

Catheter	$D_{50}\;\left(\mathrm{Gy}\right)$	$D_{m}\;\left(\mathrm{Gy}\right)$	$u\left(\%\right)$	$\Delta D\left(\%\right)$
1	4.0010	4.13	7	3.1
2	4.0004	3.75	7	−6.7
3	4.0003	3.69	8	−8.4
4	4.0016	2.41	6	−66.0

## Data Availability

Data is contained within the article.
